# Severe Clinical Course in a Patient with Congenital Amegakaryocytic Thrombocytopenia Due to a Missense Mutation of the c-MPL Gene

**DOI:** 10.4274/tjh.2013.0191

**Published:** 2015-05-08

**Authors:** İkbal Ok Bozkaya, Neşe Yaralı, Pamir Işık, Rukiye Ünsal Saç, Betül Tavil, Bahattin Tunç

**Affiliations:** 1 Ankara Children’s Hematology Oncology Hospital, Clinic of Pediatric Hematology, Ankara, Turkey

**Keywords:** Congenital amegakaryocytic thrombocytopenia, Thrombopoietin, c-MPL, Homozygous missense mutation, c-MPL Tryp154Arg, Amino acid change

## Abstract

Congenital amegakaryocytic thrombocytopenia (CAMT) generally begins at birth with severe thrombocytopenia and progresses to pancytopenia. It is caused by mutations in the thrombopoietin receptor gene, the myeloproliferative leukemia virus oncogene (c-MPL). The association between CAMT and c-MPL mutation type has been reported in the literature. Patients with CAMT have been categorized according to their clinical symptoms caused by different mutations. Missense mutations of c-MPL have been classified as type II and these patients have delayed onset of bone marrow failure compared to type I patients. Here we present a girl with severe clinical course of CAMT II having a missense mutation in exon 4 of the c-MPL gene who was admitted to our hospital with intracranial hemorrhage during the newborn period.

## Abstract (tr)

Konjenital amegakaryositik trombositopeni (KAMT) genellikle, doğumda ağır trombositopeni ile başlar ve pansitopeniye ilerler. Hastalığın nedeni trombopoetin reseptör geni olan myeloproliferatif lösemi virüs onkogenindeki (c-MPL) mutasyon olup, literatürde KAMT kliniği ile c-MPL mutasyon tipleri arasında ilişki bildirilmiş, hastalar farklı mutasyonların neden olduğu klinik belirtilere göre sınıflandırılmışlardır. c-MPL’nin yanlış anlamlı mutasyonları tip 2 olarak sınıflanmıştır. Bu hastalar tip 1 hastalar ile karşılaştırıldığında kemik iliği yetmezliğinin daha geç başladığı bildirilmiştir. Burada, yenidoğan döneminde intrakranial kanama ile hastanemize başvuran c-MPL geninde ekzon 4’de yanlış anlamlı mutasyonu olan KAMTII tanılı ağır klinik seyirli bir kız sunulmuştur.

## INTRODUCTION

Congenital amegakaryocytic thrombocytopenia (CAMT), one of the autosomal recessive hereditary bone marrow deficiency syndromes, generally begins at birth with severe thrombocytopenia and progresses to pancytopenia [1]. Other syndromes presenting with severe thrombocytopenia at birth are thrombocytopenia with the absence of the radius bone, amegakaryocytic thrombocytopenia with radioulnar synostosis, and Paris-Trousseau syndrome. The molecular pathophysiology of CAMT was explained after the discovery of thrombopoietin (TPO) and its receptor, namely the myeloproliferative leukemia virus oncogene (c-MPL) [2]. The c-MPL gene consists of 12 exons located in the 1p34 locus. In the literature, 41 mutations in the c-MPL gene were defined [1,3,4]. Most CAMT patients have homozygous or compound heterozygous mutations in the c-MPL gene, which lead to absent or impaired reactivity to TPO [5]. Ballmaier et al. published a series of CAMT cases in 2001 and suggested 2 groups of genotype-phenotype features. CAMT I is a severe course of the disease with early development of pancytopenia due to a complete loss of function of the TPO receptor. However, CAMT II patients may show a transient increase of platelet counts during the first year of life with missense mutations of the c-MPL gene [2]. Clinical highlights of CAMT are severe thrombocytopenia secondary to ineffective thrombopoiesis and bone marrow deficiency due to a failure of early hematopoietic progenitors. This indicates the critical role of TPO in both megakaryocytopoiesis and maintenance of stem cells [4]. Thrombocytopenia or associated symptoms appear in 70% of cases at birth and 90% of cases in the first year of life [1]. 

We hereby present a girl with a severe clinical course of CAMT II having a missense mutation in exon 4 of the c-MPL gene who was admitted to our hospital with intracranial hemorrhage (ICH) during the newborn period. Informed consent was obtained.

## CASE PRESENTATION

**Figure 1:**
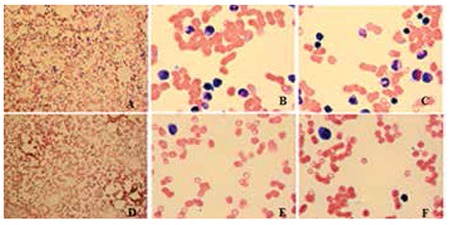
Progression of bone marrow failure in a child with CAMT. A, B, C- Bone marrow aspiration performed at 2 years of age showed no megakaryocytes in a cellular particle with erythroid and myeloid precursors without dysplasia (100x, 1000x, 1000x, respectively). D, E, F- Bone marrow aspirate showed very hypocellular results with few lymphocytes at 2.5 years of age (100x, 100x, 1000x, respectively).

A 2-day-old girl was admitted to our hospital with petechiae and purpura. Her past medical history revealed that she had ICH at the 28th week of gestation as detected by fetal ultrasonography. No fetal intervention was applied. She was born by cesarean section at the 38th week of gestation with a birth weight of 2750 g. Her parents were first cousins. On physical examination, no congenital abnormalities were revealed. Cranial USG revealed ICH and head computed tomography showed a severe parenchymal hemorrhage on her first day of life. Initial laboratory studies revealed hemoglobin (Hb) of 134 g/L, white blood cell (WBC) count of 10.3x109/L, platelet (PLT) count of 6x109/L, and mean platelet volume of 6 fL. Her mother’s platelet count was normal. Bone marrow aspiration disclosed absence of megakaryocytes. She was diagnosed with CAMT. Analysis of the patient’s TPO revealed a very high level (564 pg/mL; normal range: 120±76 pg/mL); however, her parents’ TPO levels were below 32 pg/mL. Molecular analysis disclosed a homozygous missense mutation in exon 4, which causes a change in arginine instead of tryptophan at the 154th amino acid position. The same heterozygote mutation was detected in her mother, father, and 2 siblings. However, she was lost to follow-up for 2 years. Two years later, she was admitted to our intensive care unit with gastrointestinal bleeding. At that time, laboratory analysis revealed Hb of 47 g/L, WBC count of 8.2x109/L, absolute neutrophil count of 3.7x109/L, and PLT count of 5x109/L. Her bone marrow aspiration smears revealed a decline in bone marrow cellularity and erythroid and myeloid cells, in addition to a decreased number of megakaryocytes (Figure 1). Bone marrow biopsy showed 25% cellularity and a few megakaryocytes were confirmed by CD61 staining. During the follow-up period, an intraventricular shunt was placed in order to treat increased intracranial pressure due to ICH in the prenatal period. During her follow-up for the last 2 years, she has had pancytopenia. The search for a bone marrow donor was unsuccessful among both family and unrelated donors.

## DISCUSSION AND REVIEW OF THE LITERATURE

CAMT is a rare disorder characterized by the lack of megakaryocytic progenitors in the bone marrow. Patients with CAMT are categorized according to their clinical symptoms caused by different mutations [6]. The first group, CAMT I, has total loss of TPO receptors due to homozygous nonsense mutations, deletions, and frame shift mutations, and its clinical course is more severe than that of the other group. Thrombocytopenia is more serious and persistent and pancytopenia begins in the first year of life. In the second group, CAMT II, there are partially functioning TPO receptors due to homozygous or compound heterozygous missense mutations and the clinical features are mild. Transient increases may be seen in platelet counts during the course of the disease. In this group, pancytopenia is not as frequent as in patients with CAMT I [2,7,8,9]. Ballmaier and Germeshausen reported 41 different mutations detected in 58 thrombocytopenic patients with CAMT [1]. More than 60% of these mutations were located in exons 2 or 3. Missense mutations in exon 4 were obtained in 8 patients, whereas a nonsense mutation was found in only 1 patient. Our patient was found to bear a homozygous missense mutation in exon 4. Cases with missense mutations in exon 4 are categorized as CAMT II and the clinical course is expected to be milder than that of patients with CAMT I. However, our patient had a relatively severe clinical course, presenting with ICH in utero and platelet counts that did not increase during follow-up. Pancytopenia developed at the early age of 2 years. Measurement of plasma TPO levels is very important in the evaluation of CAMT, but the TPO level could not be measured routinely. TPO is produced in the liver and removed from circulation by receptor-mediated uptake and degradation [10,11]. Most TPO receptors in megakaryocytes and thrombocytes are expressed by the c-MPL gene [4]. Thrombopoiesis is remarkably decreased in patients with CAMT because of the absence of functional c-MPL expression. As a result, the plasma TPO level is frequently ≥10 times higher in children with CAMT than in controls [2]. However, TPO level is normal or slightly increased in patients with immune thrombocytopenia in which platelet destruction is the main cause of thrombocytopenia [12]. CAMT patients usually show 10- to 50-fold elevated TPO levels compared to normal donors. In our patient, the TPO level was higher than normal, but not as high as is observed in patients with CAMT I. However, her platelet count was persistently below 20x109/L and she developed pancytopenia in the very early period, which is not expected in this group of patients. The genotype-phenotype discrepancy despite relatively low levels of TPO in our patient could be caused by factors related to the host or additional genetic alterations that could not be observed during routine analysis.

In conclusion, CAMT is a rare cause of thrombocytopenia in childhood. Children suspected of CAMT should be analyzed for mutations in c-MPL, confirmative for the diagnosis of CAMT. The role of the clinical phenotypes of CAMT I and CAMT II is not yet clear, especially regarding the development of bone marrow failure and the influence of other regulatory genes and epigenetic factors on the phenotype of CAMT.
